# Selective bioactive effects of *Anisosciadium lanatum* Boiss. [Apiaceae] essential oil: GC-MS profiling coupled with *in Vitro* and *in silico* screening

**DOI:** 10.3389/fphar.2026.1772426

**Published:** 2026-05-12

**Authors:** Malek Besbes, Saoussen Jilani, Assia Hamdi, Amal Dbeibia, Siwar Rich, Mabrouk Horchani, Wasimah B. Al-Shammari, Dalal AlArdan, Abeer Ayed Alshammari, Mouna Ghorbel, Ramzi Hadj Lajimi, Hichem Ben Jannet, Walid Ben Selma

**Affiliations:** 1 Department of Biology, College of Science, University of Hail, Ha’il, Saudi Arabia; 2 Laboratory of Chemical, Pharmaceutical and Pharmacological Development of Drugs, Faculty of Pharmacy, University of Monastir, Monastir, Tunisia; 3 Laboratory of Physiopathology, Food and Biomolecules, LR17ES03, Higher Institute of Biotechnology of Sidi Thabet, University of Manouba, Ariana, Tunisia; 4 Faculty of Dentistry, Laboratory of Research on Biologically Compatible Compounds, University of Monastir, Monastir, Tunisia; 5 Laboratory of Heterocyclic Chemistry, Natural Products and Reactivity (LR11Es39), Medicinal Chemistry and Natural Products, Faculty of Science of Monastir, University of Monastir, Avenue of Environment, Monastir, Tunisia; 6 Department of Chemistry, College of Science, University of Ha’il, Ha’il, Saudi Arabia; 7 Laboratory of Analysis, Treatment and Valorization of Environmental Pollutants and Products, Faculty of Pharmacy, Monastir University, Monastir, Tunisia

**Keywords:** ADME prediction, Anisosciadium lanatum Boiss, antibacterial activity, chemical scavenging capacity, enzyme inhibition, essential oil, molecular docking

## Abstract

**Background:**

Essential oils are a rich source of secondary metabolites with diverse chemical and functional properties.

**Objective:**

This study aimed to analyze the chemical composition of *Anisosciadium lanatum* Boiss. [Apiaceae] essential oil and to evaluate its bioactivity profile, including cytotoxicity, enzymatic inhibition (α-amylase and lipoxygenase), and antibacterial effects, as well as its chemical radical-scavenging capacity. *In silico* simulations, including molecular docking and ADME profiling, were performed to uncover the molecular basis of the observed effects and chemical interactions.

**Methods:**

The chemical composition was determined via Gas Chromatography-Mass Spectrometry (GC-MS). The antioxidant capacity was evaluated using 2,2-diphenyl-1-picrylhydrazyl (DPPH) radical scavenging, *β*-Carotene bleaching, and 2,2′-azino-bis (3-ethylbenzothiazoline-6-sulfonic acid) (ABTS) radical cation assays. Biological evaluation included a cell-based *in vitro* cytotoxicity assay (MTT), enzymatic inhibition assays (*α*-amylase and lipoxygenase), and antibacterial testing through MIC/MBC determination.

**Results:**

GC-MS analysis identified isopulegol (22.39%), longifolene (19.73%), and *β*-asarone (11.27%) as the major metabolites. The oil demonstrated a high chemical scavenging capacity with SC_50_ values of 0.019 ± 0.010 mg/mL (DPPH), 0.041 ± 0.040 mg/mL (ABTS^+^), and 0.094 ± 0.010 mg/mL (*β*-carotene bleaching). In biological models, cytotoxicity in HEK-293 cells revealed an IC_50_ value of 0.060 ± 0.010 mg/mL, indicating moderate activity. Consequently, further studies using primary human cells or *in vivo* models are warranted to comprehensively evaluate its safety and toxicological profile. Notably, the oil exhibited enzyme inhibition against *α*-amylase (IC_50_ = 0.010 ± 0.010 mg/mL), indicating preliminary glucose-modulating potential, and moderately inhibited lipoxygenase (IC_50_ = 0.101 ± 0.020 mg/mL). Antibacterial testing revealed a significant bactericidal effect against *Staphylococcus aureus*. *In silico* analysis provided supportive computational insights: alloaromadendrene showed favorable predicted binding to human erythrocyte catalase (–7.9 kcal/mol) and *S. aureus* dehydrosqualene synthase (–8.9 kcal/mol), while longifolene exhibited promising docking scores for *α*-amylase (–7.0 kcal/mol) and lipoxygenase (–6.3 kcal/mol). Additionally, ADME profiling suggested potentially favorable pharmacokinetic properties for the principal metabolites.

**Conclusion:**

Overall, *A. lanatum* essential oil represents a potential source of bioactive metabolites. While it shows potent chemical-scavenging capacity, its enzyme-inhibitory and antibacterial effects suggest potential bioactive properties that require further investigation in advanced biological models.

## 1 Introduction

Cells naturally generate reactive oxygen species (ROS) as part of normal metabolism and in response to stress. When the accumulation of these molecules exceeds the body’s capacity to neutralize them, oxidative stress develops, resulting in damage to DNA, cell membranes, and proteins.

Such imbalances contribute to metabolic disorders, including type 2 diabetes, by impairing insulin signaling and disrupting glucose homeostasis through oxidative stress–mediated mechanisms (Chen et al., 2025; Yesupatham and Saraswathy, 2025).

Simultaneously, the rapid rise of multidrug-resistant bacteria presents a global health challenge, decreasing the effectiveness of current antibiotics and raising the risk of severe infections (WHO, 2024; Nazir et al., 2025). Plant-derived essential oils have gained attention because of their diverse bioactive metabolites, small molecular size, and strong penetration ability, which enable antioxidant, antimicrobial, and anti-inflammatory effects. These natural metabolites are generally recognized as safe, and some metabolites have been approved for human use as food additives or therapeutic agents (Redondo-Blanco et al., 2020; US Food and Drug Administration, 2024; Ullah et al., 2025). Previous research from our group and others has emphasized the antibacterial potential of essential oils from medicinal plants against various multidrug-resistant pathogens (Ben Selma et al., 2024a; Ben Selma et al., 2025; Iskander et al., 2025; Jilani et al., 2025; Jilani et al., 2026).

The Apiaceae family, comprising over 3,700 species, is rich in specialized metabolites such as terpenoids, flavonoids, saponins, and coumarins. Many of these metabolites exhibit significant pharmacological activities, including hepatoprotective, antitumor, antidiabetic, and antimicrobial effects (Khalil et al., 2022; Matar et al., 2022; Bag et al., 2024). Several members of this family have a long history of traditional use as botanical drugs for managing metabolic disorders, including modulation of blood glucose levels (Amiri and Joharchi, 2016; Arraji et al., 2024). Within this diverse family, the genus *Anisosciadium* has traditionally been used to treat various ailments, including skin disorders, fever, gastrointestinal disturbances, and other conditions commonly associated with inflammatory and oxidative processes (Besbes et al., 2025b). Specifically, *Anisosciadium lanatum* Boiss. [Apiaceae] is an aromatic botanical drug found across the Arabian Peninsula and has traditional uses in Saudi Arabia for treating skin conditions and as a dietary plant (Sher and Aldosari, 2013; El-Sayed et al., 2017; Mandaville, 2019). Previous studies have also reported its antioxidant capacity and anti-proliferative effects, as well as the presence of bioactive guaiane sesquiterpenes with anti-mutagenic properties (Mahmoud and El-Sayed, 2019).

However, no studies have examined the essential oil metabolites of *A. lanatum* from the Hail region. To address this gap, the present study extracted and characterized these metabolites and evaluated their chemical antioxidant capacities (via DPPH, ABTS, and *β*-carotene bleaching assays), alongside their bioactive effects, including antibacterial properties, *α*-amylase and lipoxygenase enzymatic inhibition, as well as *in vitro* cytotoxicity using HEK-293 cells. GC–MS metabolite profiling, supported by molecular docking and molecular dynamics simulations, was conducted to validate and interpret the experimental findings. This integrated approach provides new insights into the bioactive potential of this botanical drug within a broader ethnopharmacological and chemotaxonomic context.

## 2 Materials and methods

### 2.1 Plant collection

The aerial parts of *Anisosciadium lanatum* Boiss. [Apiaceae] were collected from the Farms of Simira governorate, south of Hail, Kingdom of Saudi Arabia, in March 2023. The species was taxonomically identified by Dr. Belsem Marzouk at the Faculty of Pharmacy, Monastir University, Tunisia, and its taxonomic status was validated using the Plants of the World Online (POWO) database (POWO, 2026). A Voucher specimen (Al - 83) was prepared for the species and deposited in the herbarium of the laboratory of biology in the College of Sciences (Simira branch–Hail University, Saudi Arabia).

### 2.2 Extraction

The essential oil was extracted from the fresh aerial parts of *A. lanatum* via hydrodistillation using a Clevenger-type apparatus, as previously described (Besbes et al., 2012). In brief, 700 g of the aerial part was placed in a 2 L round-bottom flask containing 1.5 L of distilled water. The mixture was heated using a digital heating mantle and maintained at 100 °C ± 2 °C for 3 h. The resulting essential oil was dried over anhydrous sodium sulfate, transferred to amber glass vials to minimize light-induced degradation, and stored at 4 °C until further analysis. The oil yield based on the fresh weight of the extract was calculated.

The extraction process and taxonomic identification were performed in accordance with the ConPhyMP guidelines (Heinrich et al., 2022). Detailed information is provided in Supplementary Table S1 and Supplementary Table 2a.

### 2.3 Phytochemical and analytical characterization

#### 2.3.1 Gas chromatography–mass spectrometry analysis

The phytochemical composition of *A. lanatum* essential oil was studied through Gas Chromatography-Mass Spectrometry (GC–MS) analysis based on our established method (Besbes et al., 2025a). The PerkinElmer Clarus 600 T system operated with a single quadrupole mass spectrometer for analysis. The Elite-5MS capillary column (30 m × 0.25 mm i.d., 0.25 µm film thickness) served as the separation column. The carrier gas used for separation was helium at 1.0 mL/min with 99.999% purity. The system accepted 1 µL of sample through a split injection method at a 1:40 ratio while maintaining the injector temperature at 250 °C. The oven temperature started at 40 °C for 1 min before it rose to 150 °C at 10 °C/min and then reached 300 °C at the same rate before maintaining that temperature for 3 min. The transfer line operated at 220 °C while the ion source maintained a temperature of 200 °C. The mass spectrometer operated at 70 eV electron ionization to scan m/z values from 40 to 618 while the system waited 3.0 min before starting data collection. The identification of volatile metabolites was performed by comparing their mass spectra with those stored in the Wiley sixth edition and NIST mass spectral libraries, as well as with published data (Adams, 2017). Retention indices (RI) reported in the literature, based on C6–C26 n-alkane standards, were used as supportive information to aid metabolite identification, particularly for distinguishing isomeric terpenes.

#### 2.3.2 DPPH radical scavenging capacity

The DPPH radical scavenging capacity was evaluated according to Hlila et al. (2016). This analytical tool assesses the chemical electron-donating ability of the oil. Briefly, 50 µL of the sample (0.0024, 0.0048, 0.009, 0.019, 0.039, 0.078, 0.156, 0.312, 0.625, 1.25, 2.5, and 5 mg/mL) was added to 200 µL of the freshly prepared DPPH solution (4 × 10^−3^% in methanol). After 30 min of incubation in the dark at 37 °C, the absorbance was measured at 517 nm using a UV spectrophotometer. All experiments were performed in triplicate. The results were expressed as SC_50_ values (mg/mL), representing the concentration required to scavenge 50% of the DPPH radical.

#### 2.3.3 ABTS radical cation scavenging capacity

The ABTS^+・^radical scavenging capacity was determined according to the method previously described by Hlila et al. (2015). This assay measures the chemical reduction of the ABTS radical cation by the oil’s metabolites, resulting in a decrease in absorbance at 734 nm using a UV spectrophotometer. The essential oil was dissolved in ethanol to obtain the following concentrations: 0.0024, 0.0048, 0.009, 0.019, 0.039, 0.078, 0.156, 0.312, 0.625, 1.25, 2.5, and 5 mg/mL. All measurements were carried out in triplicate. The results were expressed as SC_50_ values (mg/mL).

#### 2.3.4 *β*-Carotene-Linoleic acid bleaching assay

The *β*-carotene bleaching assay was performed to evaluate the chemical inhibition of lipid peroxidation (Hlila et al., 2015). Briefly, 2 mL of *β*-carotene solution was mixed with 200 µL of Tween 20 and 20 µL of linoleic acid. After solvent evaporation, 50 mL of distilled water was added with vigorous shaking to form a *β*-carotene-linoleic acid emulsion. Aliquots of 5 mL of the emulsion were mixed with 500 µL of sample solutions (0.0024–5 mg/mL). The mixtures were incubated in a water bath at 50 °C for 2 h. Absorbance was measured at 470 nm using a UV spectrophotometer. All experiments were performed in triplicate. Chemical antioxidant capacity (CAC%) was calculated using the following equation:

$$\left(\text{CAC}\%\right)=\left(\beta -\text{carotene content after }2\;h\;/\text{ initial }\beta -\text{carotene content}\right)\times 100.$$

and the SC_50_ value (mg/mL) was then determined. This assay serves as an analytical indicator of the oil’s redox properties in an emulsified system.

### 2.4 Cytotoxicity evaluation

#### 2.4.1 Cell culture

Human embryonic kidney HEK-293 cells were obtained from the American type culture collection (ATCC) and were cultured at 37 °C and 5% CO2 humidity in Dulbecco’s Modified Eagle’s Medium (DMEM), supplemented with 10% FBS, 100 units/mL penicillin (pen) and 2 mM L-glutamine. HEK-293 cells exhibit high efficiency in transfection experiments.

#### 2.4.2 *In vitro* cytotoxicity assay (MTT)

The impact of *A. lanatum* essential oil on the viability of HEK-293 cells was assessed using an MTT [3-(4,5-dimethylthiazol-2-yl)-2,5-diphenyltetrazolium bromide] test, as previously described (Ayadi et al., 2023). Briefly, HEK-293 cells were plated in 96-well plates, at a density of 1.5 × 10^4^ cells/well for 24 h in DMEM, supplemented with 10% Fetal Bovine Serum (FBS), 2 mM L-glutamine, and 100 units/mL penicillin. The oil was dissolved in dimethyl sulfoxide (DMSO) and added to the wells at concentrations ranging from 0.039 to 5 mg/mL (via two-folds serial dilutions). Following 24 h of incubation at 37 °C and 5% CO_2_, 0.5 mg/mL MTT reagent was added for 3–4 h. The resulting formazan crystals were dissolved in 100% isopropanol, and the absorbance was recorded at 570 nm using a microplate reader (EnSpire, PerkinElmer, Beaconsfield, United Kingdom). Doxorubicin was employed as a positive control.

### 2.5 Enzymatic *α*-amylase inhibition assay

The α-amylase enzymatic inhibition was evaluated according to previously reported methods with minor modifications (Ali et al., 2006; Souza et al., 2012). This enzyme assay was used to determine the inhibitory effect of the essential oil on α-amylase activity. The reaction mixture in a 96-well plate consisted of 100 mM phosphate buffer (pH 6.8), α-amylase solution (2 U/mL), and various concentrations of the oil (0.0024–5 mg/mL). The mixture was pre-incubated at 37 °C for 20 min. The reaction was initiated by adding 1% soluble starch solution and incubated at 37 °C for 30 min. The reaction was terminated by adding 3,5-dinitrosalicylic acid (DNS) reagent, followed by heating in a boiling water bath for 20 min. After cooling, absorbance was measured at 540 nm using a microplate spectrophotometer (TECAN Infinite M200 Pro, Männedorf, Switzerland). Acarbose was used as a positive control, while the negative control contained all reagents except the oil. All assays were performed in triplicate. The percentage of enzymatic inhibition was calculated as follows:

$$\%\text{Inhibition}=\left[\left(Ac-As\right)\;/Ac\right]\times 100$$

Where, *A*c is the absorbance of the control and *A*s is the absorbance of the sample.

The IC_50_ values were determined from the inhibition curves.

### 2.6 Enzymatic lipoxygenase inhibition assay

The anti-lipoxygenase effect was evaluated based on the enzymatic inhibition of soybean lipoxygenase-catalyzed oxidation of linoleic acid (Eshwarappa et al., 2016; Nile et al., 2020). Various concentrations of *A. lanatum* essential oil (0.039–5 mg/mL) were dissolved in 0.25 mL of 2 M borate buffer (pH 9.0). This solution was mixed with 0.25 mL of soybean lipoxygenase enzyme solution and incubated at 25 °C for 5 min. The reaction was initiated by adding 1 mL of linoleic acid solution. The increase in absorbance was measured at 234 nm using UV spectrophotometer. Dexamethasone (60 μg/mL) was used as a reference control. All experiments were performed in triplicate. The percentage of enzymatic inhibition was calculated as follows:

$$\%\text{Inhibition}=\left[\left(Ac-As\right)\;/Ac\right]\times 100$$

Where, *A*c is the absorbance of the control and *A*s is the absorbance of the sample.

The IC_50_ values were determined from the inhibition curves.

### 2.7 Antibacterial potential

#### 2.7.1 Bacterial strains

The growth-inhibitory effects of *A. lanatum* essential oil were evaluated against three Gram-negative strains: *Escherichia coli* (ATCC 25922), *Salmonella enterica* (ATCC 43972), and *Pseudomonas aeruginosa* (ATCC 9027), and three Gram-positive strains: *Staphylococcus aureus* (ATCC 25923), *Listeria monocytogenes* (ATCC 19117), and *Bacillus cereus* (ATCC 14579).

#### 2.7.2 Preparation of stock solutions

The *A. lanatum* essential oil stock solutions were prepared by dissolving the oil in DMSO (Sigma-Aldrich, United States) to achieve a concentration of 100 mg/mL. Working solutions (0.078–10 mg/mL) were then prepared using two-fold serial dilutions in Mueller–Hinton II cation-adjusted broth (Sigma-Aldrich, United States). The final DMSO concentration in all assays was maintained below 0.05% (v/v), a level previously shown to have no inhibitory effect on bacterial growth (Cirino et al., 2023).

#### 2.7.3 Bacterial inoculum

Bacterial inocula were prepared according to CLSI guidelines (2019). Overnight cultures were adjusted to a 0.5 McFarland standard (approximately 1 × 10^8^ CFU/mL) and subsequently diluted 1:1000 to achieve a final test inoculum of 1 × 10^5^ CFU/mL for the microdilution assays.

#### 2.7.4 Minimum Inhibitory Concentration (MIC) assay

The MIC of *A. lanatum* essential oil was determined using the broth microdilution method in accordance with standard protocols (Clinical and Laboratory Standards Institute (CLSI), 2019; EUCAST, 2024). Briefly, the oil was diluted in sterile 96-well microplates (SARSTEDT AG & Co. KG, Numbrecht, Germany) to final concentrations ranging from 0.078 mg/mL to 10 mg/mL. The bacterial suspension (1 × 10^5^ CFU/mL) was added to each well (total volume 100 µL). To assess metabolic viability, 10 µL of resazurin solution (0.1 mg/mL) was added as a redox indicator. The plates were incubated at 37 °C for 24 h (Edziri et al., 2012; Hamdi et al., 2023; Ben Selma et al., 2024b). The MIC was defined as the lowest concentration that visibly inhibited bacterial growth. Sterility controls (broth only) and growth controls (broth with inoculum, without oil) were included in each assay to ensure medium sterility and bacterial viability, respectively. Additionally, a solvent control (0.05% v/v DMSO) was performed to ensure no interference with microbial growth.

#### 2.7.5 Minimum Bactericidal Concentration (MBC) assay

To determine the MBC, 10 µL aliquots from wells showing no visible growth were subcultured onto Mueller–Hinton agar plates (Sigma-Aldrich, United States) and incubated at 37 °C for 24 h. The MBC was defined as the lowest concentration resulting in no bacterial colony growth (Ben Selma et al., 2025; Jilani et al., 2025; Besbes et al., 2026). The *A. lanatum* essential oil was classified as bactericidal if the MBC/MIC ratio was ≤4 and bacteriostatic if the ratio was >4 (Pankey and Sabath, 2004; Hamdi et al., 2023; Besbes et al., 2025b).

### 2.8 *In silico* molecular docking analysis

The molecular docking simulations were conducted using the AutoDock 4.2 software package (Trott and Olson, 2010). The crystal structures of the target proteins were retrieved from the Research Collaborator for Structural Bioinformatics (RCSB) Protein Data Bank (https://www.rcsb.org/): Human erythrocyte catalase (PDB ID: 1DGH), (Putnam et al., 2000), Human pancreatic α-amylase (PDB ID: 3BAJ) (Maurus et al., 2008), human 5-lipoxygenase (PDB ID: 3V99) (Gilbert et al., 2012), and DNA gyrase catalytic core from *Staphylococcus aureus* (PDB ID: 2ZCQ) (Liu et al., 2008). Prior to docking, protein structures were prepared by removing water molecules, adding missing hydrogen atoms, and assigning Gasteiger charges. The AutoDock Tools software produced Protein Data Bank Q partial charge T Atom Type (PDBQT) format files which contained both ligand and protein data while AutoGrid performed pre-calculated grid map optimization for better computational performance. The ACD 3D Viewer software (http://www.filefacts.com/acd3d-viewer-freeware-info) was used to perform three-dimensional geometry optimization for all ligands. The analysis of protein–ligand interactions and docking results used Discovery Studio 2017 R2 (https://www.3dsbiovia.com/products/collaborative-science/biovia-discovery-studio/) and PyMOL 0.99rc6 (Delano, 2002).

### 2.9 Pharmacokinetic and ADME properties

The pharmacokinetic profiles and drug-likeness properties of the major metabolites identified in *A. lanatum* essential oil were predicted using the SwissADME online platform (http://www.swissadme.ch/). Key parameters related to ADME (absorption, distribution, metabolism, and excretion) were evaluated to assess the potential bioavailability and medicinal suitability of the identified metabolites from this botanical drug.

### 2.10 Statistical analysis

All experiments were conducted with at least three independent replicates (n = 3), and results are presented as mean ± standard deviation (SD). One-way ANOVA was applied to compare group means, followed by Duncan’s multiple range test for pairwise comparisons. Exact p-values are provided in the tables or figure legends. The SC_50_ values for chemical antioxidant assays (DPPH, ABTS, and *β*-carotene bleaching) and IC_50_ values for cytotoxic and enzymatic inhibition assays were calculated from dose–response curves using linear regression, with goodness-of-fit assessed via the coefficient of determination (*R* ^2^). Statistical comparisons with reference standards were performed where applicable, and all analyses were conducted using IBM SPSS Statistics software (version 22, IBM Corp., Armonk, NY, United States), with *p* ≤ 0.05 considered statistically significant.

## 3 Results and discussion

### 3.1 Phytochemical and analytical characterization

#### 3.1.1 Gas chromatography–mass spectrometry analysis

**FIGURE 1:**
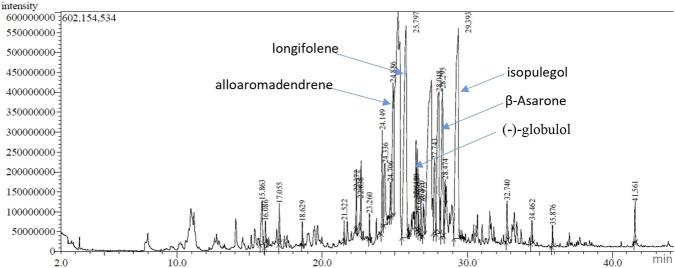
Chromatogram of *Anisosciadium lanatum* Boiss. essential oil by GC-MS method.

The hydrodistillation of the fresh aerial part of *A*. *lanatum* yielded a yellow-colored essential oil with a yield of 0.035%. GC–MS analysis identified twenty-two metabolites, representing 93.44% of the total volatile metabolites (Table 1). The oil was predominantly composed of sesquiterpene hydrocarbons (34.08%) and oxygenated monoterpenes (27.72%), followed by oxygenated sesquiterpenes (18.92%) and phenylpropanoids (11.27%), while non-terpene derivatives accounted for only a minor fraction (1.45%). Among the identified metabolites, isopulegol (22.39%) and longifolene (19.73%) were the major metabolites, followed by *β*-asarone (11.27%), globulol (8.55%), and alloaromadendrene (7.06%) (Figure 1). *β*-asarone was detected at a moderate level (11.27%). Previous studies have reported potential genotoxic and carcinogenic effects under certain experimental conditions (Chellian et al., 2017). The simultaneous predominance of oxygenated and hydrocarbon terpenes suggests a balanced chemical profile composition that can contribute to potential synergistic biological activities of the oil. Compared with previous studies, thirteen metabolites were identified for the first time in the *Anisosciadium* genus, including isopulegol, lingifolene, alloaromadendrene, aromadendrene, prostantherol, neodihydrocarveol, viridiflorol, γ-eudesmol, humulenol, hystrene, falcarinol, globulol, and cis-11-eicosenamide. The remaining metabolites have been previously reported in either *A. lanatum* or *A. orientale* essential oils, although in varying proportions. Al-Mazroa (2003) and Khalil et al. (2022) investigated the essential oils of *A. lanatum* collected from Riyadh (Saudi Arabia), and reported that trans-caryophyllene, *α*-humulene, *α*-terpineol, bornyl acetate, and caryophyllene oxide as common metabolites. These metabolites were also detected in the present essential oil obtained from Simira, Governorate of Hail (KSA). Furthermore, several metabolites identified in the current oil, including *β*-asarone, germacrene B, *β*-elemene, and spathulenol, have also been reported in *A. orientale* essential oil. Oxygenated monoterpenes such as *α*-terpineol and isopulegol are widely recognized for their strong antioxidant and antimicrobial activities, which may partly explain the biological activities observed in this study. Likewise, sesquiterpene hydrocarbons including longifolene and caryophyllene, have frequently been asssociated with anti-inflammatory effects. The relatively high proportion of oxygenated sesquiterpenes, such as spathulenol, caryophyllene oxide, and viridiflorol, further supports the potential pharmacological relevance of the oil due to their reported bioactivities. Overall, GC–MS analysis revealed that the essential oil possesses a chemically diverse profile dominated by biologically active terpenoids. This compositional pattern is consistent with previous reports on essential oils from related Apiaceae species, suggesting that both oxygenated monoterpenes and sesquiterpene hydrocarbons play key roles in determining the biological activities of plant extracts (Önder et al., 2024).

**TABLE 1:** Identified metabolites by GC-MS in the *Anisosciadium lanatum* Boiss. essential oil.

Peak	Metabolite name	Molecular formula	Rt (min)	RI (lit)	Area %
1	α-terpineol	C10H18O	16.086	1143	0.88
2	Neodihydrocarveol	C10H18O	17.055	1196	1.01
3	bornyl acetate	C12H20O2	18.629	1277	0.51
4	β-elemene	C15H24	21.522	1398	0.36
5	trans-caryophyllene	C15H24	22.372	1494	1.46
6	germacrene B	C15H24	23.260	1603	0.52
7	prostantherol	C15H26O	24.150	1581	2.93
8	aromadendrene	C15H24	24.336	1386	3.34
9	α-humulene	C15H24	24.706	1494	0.95
10	alloaromadendrene	C15H24	24.886	1386	7.06
11	longifolene	C15H24	25.797	1398	19.73
12	(−)-Spathulenol	C15H24O	26.488	1536	2.58
13	caryophyllene oxide	C15H24O	26.518	1507	2.59
14	(−)-globulol	C15H26O	26.635	1530	8.55
15	viridiflorol	C15H26O	26.820	1530	0.79
16	γ-eudesmol	C15H26O	27.743	1626	3.50
17	β-Asarone	C12H16O3	28.048	1568	11.27
18	humulenol	C157H24O	28.474	1762	0.91
19	isopulegol	C10H18O	29.393	1196	22.39
20	hystrene	C18H36O2	34.462	2167	0.66
21	falcarinol	C17H24O	35.876	1906	0.48
22	cis-11-Eicosenamide	C20H39NO	41.561	2427	0.97
​	Classes of metabolites	Area % (no of metabolites)			
​	Total oxygenated monoterpenes	27.72 (5)			
​	Total sesquiterpene hydrocarbons	34.08 (8)			
​	Total oxygenated sesquiterpenes	18.92 (6)			
​	Total phenylpropanoids	11.27 (1)			
​	Total non-terpene derivatives	1.45 (2)			
​	Total identified metabolites	93.44			

Rt: retention time; RI (lit): Retention indices obtained from NIST, mass spectral library and published literature (Adams, 2017).

Identification was based on comparison of mass spectra (MS) with NIST, library data.

#### 3.1.2 Chemical antioxidant capacity (analytical assays)

The chemical antioxidant capacity of *A. lanatum* essential oil was characterized using three analytical assays: DPPH, ABTS radical scavenging, and the *β*-carotene/linoleic acid bleaching system. Notably, the ABTS and *β*-carotene assays were applied to this oil for the first time. The DPPH assay was used to assess the chemical electron-donating ability of metabolites identified in the oil. The results of the chemical antioxidant capacity are summarized in Table 2. The results indicated that the essential oil exhibited a notable DPPH scavenging capacity with an SC_50_ value of 0.019 ± 0.010^b^ mg/mL, which was comparable to the analytical standard, Vitamin E (SC_50_ = 0.010 ± 0.020^a^ mg/mL). In the ABTS assay, the essential oil also exhibited considerable radical scavenging potential (SC_50_ = 0.041 ± 0.040^b^ mg/mL), slightly lower than that of Vitamin E (SC_50_ = 0.015 ± 0.030^a^ mg/mL). Likewise, in the *β*-carotene bleaching assay, which evaluates the chemical protection against lipid peroxidation in an emulsified system, the sample showed a moderate effect (SC_50_ = 0.094 ± 0.010^b^ mg/mL) compared to the reference (SC_50_ = 0.010 ± 0.020^a^ mg/mL). Overall, these findings indicate that the essential oil possesses significant analytical antioxidant potential, particularly in the DPPH and ABTS assays. The reduced efficiency observed in the *β*-carotene system may be attributed to the limited solubility of certain volatile metabolites in the lipid phase of the emulsion, a phenomenon commonly reported for essential oils. In the literature, El-Sayed et al. (2017) investigated the antioxidant properties of *A. lanatum* from Riyadh (KSA) reporting moderate scavenging in the DPPH assay (around 20% inhibition). In contrast, the essential oil from Hail evaluated in the present study exhibited a much stronger DPPH scavenging capacity. These variations in chemical performance are likely driven by differences in phytochemical composition influenced by geographical origin. Previous studies (Al-Mazroa 2003; Khalil et al., 2022) reported that *A. lanatum* oils from Riyadh were dominated by hydrocarbon terpenes such as limonene and *α*-pinene, which generally possess lower radical-scavenging capacity. In contrast, our sample from Hail was characterized by a higher proportion of oxygenated monoterpenes and sesquiterpenes, including isopulegol, prostantherol, *α*-terpineol, globulol, and spathulenol. This enhanced chemical reactivity observed in the present study may therefore be associated with the presence of oxygenated functional groups (–OH, –O–, and = O), which improve hydrogen-or electron-donating ability and facilitate free radical stabilization.

**TABLE 2:** Chemical antioxidant capacity, enzymatic inhibition, *in vitro* cytotoxic, and antibacterial screening of *Anisosciadium lanatum* Boiss. essential oil.

Assay category	Assay/Target	Result (mean ± SD) mg/mL	Reference (mg/mL)
Chemical antioxidant capacity	​	​	Vitamin E
​	DPPH radical scavenging	SC_50_ = 0.019 ± 0.010^b^	SC_50_ = 0.010 ± 0.020^a^
​	ABTS radical cation	SC_50_ = 0.041 ± 0.040^b^	SC_50_ = 0.015 ± 0.030^a^
​	β–Carotene bleaching	SC_50_ = 0.094 ± 0.010^b^	SC_50_ = 0.010 ± 0.020^a^
Enzymatic inhibition assays	​	​	Acarbose
​	α–Amylase	IC_50_ = 0.010 ± 0.010 ^a^	IC_50_ = 0.017 ± 0.001 ^b^
​	​	​	Dexamethasone
​	Lipoxygenase	IC_50_ = 0.101 ± 0.020 ^b^	IC_50_ = 0.052 ± 0.020 ^a^
​	​	​	Doxorubicin
Cytotoxic *in vitro* activity	MTT assay (HEK-293 cells)	IC_50_ = 0.060 ± 0.010 ^b^	IC_50_ = 0.005 ± 0.001 ^a^
Antibacterial screening	*Escherichia coli* ATCC 25922	MIC = 50 MBC >50	Ciprofloxacin MIC = 0.062 MBC = 0.062
​	*Salmonella enterica* ATTC 43972	MIC = 50 MBC >50	MIC = 0.125 MBC = 0.125
​	*Pseudomonas aeruginosa* ATCC 9027	MIC = 50 MBC >50	MIC = 0.125 MBC = 0.125
​	*Staphylococcus aureus* ATCC 25923	MIC = 0.78 MBC = 3.12	MIC = 0.031 MBC = 0.125
​	*Bacillus cereus* ATCC 14579	MIC >50 MBC >50	MIC = 0.031 MBC = 0.125
​	*Listeria monocytogenes* ATCC 19117	MIC = 50 MBC >50	MIC = 0.031 MBC = 0.125

SC_50_ (Half-maximal scavenging concentration) refers to the chemical antioxidant capacity in analytical assays. IC_50_ (Half-maximal inhibitory concentration) refers to the biological inhibition of enzymes and cell viability. MIC: minimum inhibitory concentration; MBC: Minimum Bactericidal Concentration. Different superscript letters in the same row indicate significant differences between the essential oil and the reference standard (*p* ≤ 0.05) according to Duncan’s multiple range test.

These findings align with Masroorbabanari et al. (2014), who reported a significantly higher SC_50_ (0.633 mg/mL) for *A. orientale* from Iran attributed to its high monoterpene hydrocarbon content (53.4%). Our results further underscore that oxygenated metabolites are the primary determinants of the chemical antioxidant strength in *Anisosciadium* species.

### 3.2 *In vitro* cytotoxicity assay (MTT)

**FIGURE 2:**
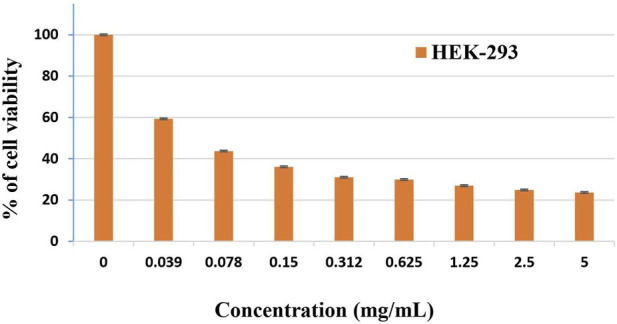
Cell viability percentage of *Anisosciadium lanatum* Boiss. essential oil on HEK-293 cells. The data are shown as the mean ± standard deviation of triplicate measurements.

The cytotoxic potential of *A*. *lanatum* essential oil was evaluated in HEK-293 cells using the MTT assay after 24 h of exposure to concentrations ranging from 0.039 to 5 mg/mL. HEK-293 cells are frequently used as a non-tumorigenic human cell model (Shaw et al., 2002; Ma et al., 2018). The essential oil induced a clear, concentration-dependent reduction in cell viability, decreasing from 59.35% at 0.039 mg/mL to 23.64% at 5 mg/mL. At concentrations ≤0.078 mg/mL, cell viability remained above 50% (Figure 2). The calculated IC_50_ was 0.060 ± 0.010^b^ mg/mL, indicating moderate *in vitro* cytotoxicity toward HEK-293 cells under the tested conditions. Doxorubicin was used as a positive control (0.005 ± 0.001^a^ mg/mL) to validate the assay performance (Table 2). These results are consistent with previous reports demonstrating comparable cytotoxic effects of *A. lanatum* essential oil toward normal fibroblast cells (NIH-3T3), with an IC_50_ of 0.052 mg/mL (Khalil et al., 2022). Nevertheless, additional investigations using primary human cells or *in vivo* models are required to further characterize its cytotoxic and toxicological profile.

### 3.3 Enzymatic *α*-amylase inhibition assay

The enzymatic *α*-amylase inhibitory activity of *A. lanatum* essential oil (IC_50_ = 0.010 ± 0.010^a^ mg/mL) indicated that this oil was more potent in inhibiting the enzyme than the standard, acarbose (IC_50_ = 0.017 ± 0.001^b^ mg/mL) (Table 2). To our knowledge, this is the first report of *α*-amylase inhibition by *A. lanatum* essential oil. While these results suggest a potential postprandial glucose-modulating effect, they should be interpreted as preliminary biochemical evidence. The phytochemical composition of this volatile oil is rich in oxygenated and hydrocarbon terpenes, which likely contribute to the observed enzymatic inhibition. Literature has shown that oxygenated monoterpenes and sesquiterpenes reveal superior inhibition of the *α*-amylase enzyme. Whereas, hydrocarbon terpenes typically indicate moderate to weak activity (Al Kury et al., 2021). Major metabolites identified in this study, such as isopulegol, longifolene, *β-*asarone, (−)-globulol, and alloaromadendrene, have not been previously evaluated for their anti-*α*-amylase potential. The observed results may be attributed to either to major or minor metabolites, or their synergistic interactions. Further studies, comprising *α*-glucosidase inhibition, insulin signaling, cellular glucose uptake, and *in vivo* evaluation, are necessary to determine the potential antidiabetic effect of this essential oil.

### 3.4 Enzymatic lipoxygenase inhibition assay

The inhibition of the soybean lipoxygenase (LOX) enzyme by the essential oil was recorded at an IC_50_ of 0.101 ± 0.020^b^ mg/mL, compared to the reference dexamethasone (0.052 ± 0.010^a^) (Table 2). Previous studies have reported higher enzymatic inhibitory activity for methanolic extracts of leaves, stems, and flowers of *A. lanatum*, with IC_50_ values of 0.005 mg/mL, 0.005 mg/mL, and 0.006 mg/mL, respectively (Matar et al., 2022). This difference in potency suggests that the polar metabolites present in the methanolic extracts may possess a stronger affinity for the lipoxygenase enzyme than the volatile metabolites identified in the essential oil. Several studies indicate that oxygenated metabolites, such as *α-*terpineol, can interact with lipoxygenase due to their polarity and ability to engage in hydrogen bonding. *In silico* studies further confirmed that both hydrocarbon and oxygenated terpenes in our essential oil can bind effectively to the enzyme’s active site (Gadnayak et al., 2022; Cherneva et al., 2025). Specifically, *β*-asarone has been reported to inhibit leukotriene production via the 5-LOX pathway in mast cells, suggesting its potential to modulate this specific enzymatic pathway (Lim et al., 2012). Overall, these findings highlight the potential of *A. lanatum* metabolites to inhibit the lipoxygenase enzyme family, although future research using cytokine assays or cellular inflammatory models is essential for a comprehensive evaluation of their broader anti-inflammatory effects.

### 3.5 Antibacterial potential

Based on the increasing prevalence of multidrug-resistant bacteria and the limited discovery of new antibiotics, infections caused by Gram-positive and Gram-negative pathogens represent a serious public health concern (Tarchouna et al., 2013; WHO, 2024). In this context, plant-derived essential oils have gained attention as potential antimicrobial agents owing to their complex mixtures of bioactive metabolites (Ben Selma et al., 2024a; Rijo, et al., 2024; Besbes et al., 2025a; Ben Selma et al., 2025; Jilani et al., 2025). The current study evaluated the antibacterial activity of *A. lanatum* essential oil against six Gram-positive and Gram-negative bacterial strains. The oil exerted selective antibacterial potential, particularly against *Staphylococcus aureus* ATCC 25923, with MIC and MBC values of 0.780 mg/mL and 3.120 mg/mL, respectively. The MBC/MIC ratio of 4, indicates a bactericidal effect. To the best of our knowledge, this is the first report describing the antibacterial activity of *A. lanatum* essential oil. Although the MIC values remain higher than those of conventional antibiotics, these findings suggest that the essential oil is more suitable as a potential lead botanical drug rather than a direct therapeutic agent.

The antibacterial activity can be associated with the phytochemical composition of the oil, which is rich in oxygenated monoterpenes and sesquiterpene hydrocarbons. Such terpenoid classes have been widely reported to exert antibacterial effects (Nazzaro et al., 2013; Rijo et al., 2024). Oxygenated sesquiterpenes in particular may interact with bacterial membranes due to their lipophilic character, while functional groups such as alcohol moieties can contribute to protein denaturation and cellular dysfunction (Muilu-Mäkelä et al., 2022). Supporting this, (−)-longifolene and isopulegol have demonstrated activity against *S. aureus* in previous studies (Naigre et al., 1996; Schmidt et al., 2010). Additionally, essential oils rich in *β*-asarone have shown membrane-disruptive antibacterial effects against *S. aureus* (Al-Mijalli et al., 2025). However, the relatively low activity reported for globulol (Mulyaningsih et al., 2010) suggests that the overall bioactivity likely results from synergistic interactions among multiple metabolites rather than from a single dominant metabolite.

No inhibitory effect was observed against the three Gram-negative strains tested. This selective activity may be partly explained by structural differences in bacterial cell envelopes, particularly the presence of an outer membrane containing lipopolysaccharides (LPS), which restricts the penetration of hydrophobic metabolites (Nazzaro et al., 2013). Moreover, outer membrane impermeability alone does not fully account for the lack of activity. The efficacy of essential oils strongly depends on the chemical nature, polarity balance, and functional groups of their major metabolites. In the present study, the predominance of non-phenolic terpenoids and the absence of strongly phenolic metabolites such as thymol or carvacrol known for their promising membrane-disruptive effects may contribute to the limited activity against Gram-negative bacteria. Therefore, the observed selectivity toward Gram-positive strains likely reflects both structural bacterial differences and the specific phytochemical profile of the oil. Overall, *A. lanatum* essential oil demonstrates selective bactericidal potential against Gram-positive bacteria. Although the activity is moderate, these findings highlight its relevance as a promising source of antibacterial lead molecules. Future investigations, including fractionation studies and evaluation of synergistic interactions, are warranted to better elucidate its therapeutic potential.

### 3.6 *In silico* molecular docking analysis

Molecular docking is an established *in silico* structure-based technique widely used in drug discovery (Pinzi and Rastelli, 2019). However, this modeling approach suggests a potential binding mode and should not be interpreted as definitive proof of the proposed mechanism. Motivated by this, *in silico* docking was employed herein to predict the possible mechanisms of action correlating with the recorded antioxidant, enzymatic (*α*–amylase, and LOX), and antibacterial potentials. The analysis focused on the major metabolites: alloaromadendrene, longifolene, (−)-globulol, *β*-Asarone, and isopulegol.

#### 3.6.1 Molecular docking against human erythrocyte catalase

**FIGURE 3:**
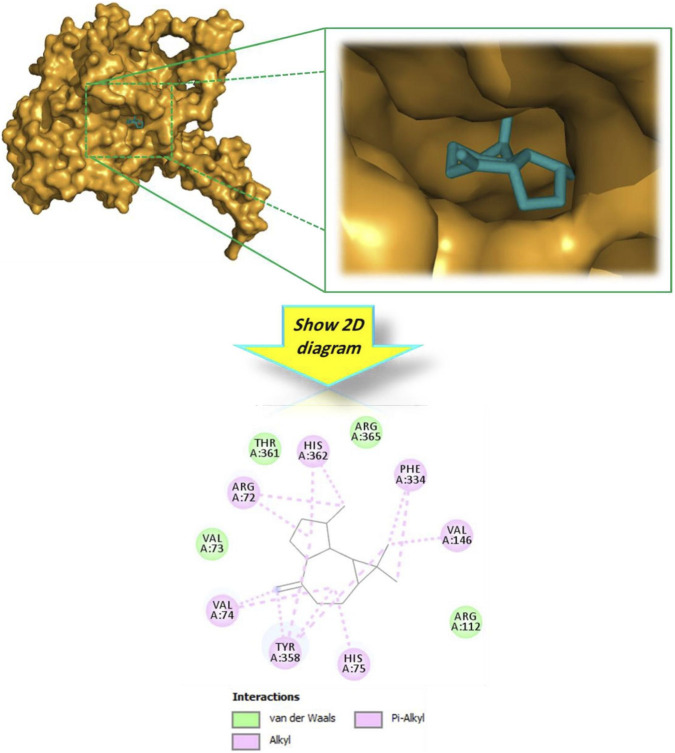
3D and 2D interactions model of the most active metabolite ‘alloaromadendrene’ within the active site of ‘human erythrocyte catalase’ (PDB ID: 1DGH).

**FIGURE 4:**
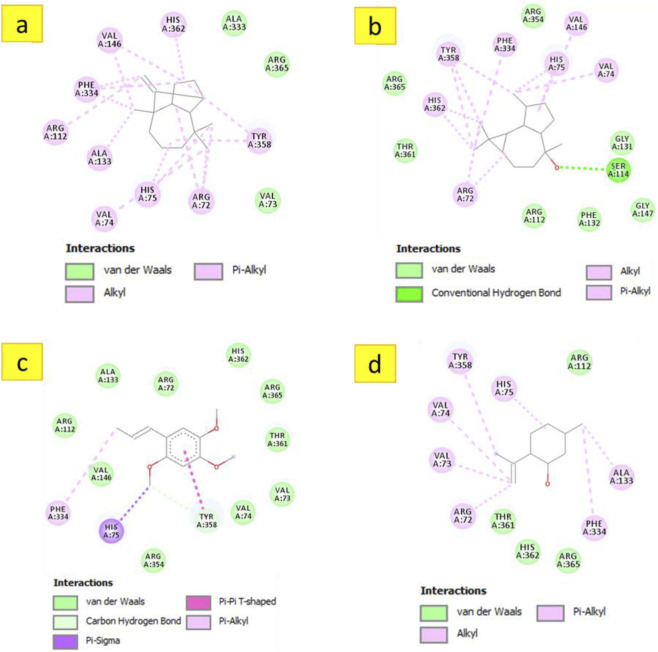
2D model of different interactions formed by the docked metabolites: longifolene **(a)**, (−)-globulol **(b)**, ẞ-asarone **(c)**, and isopulegol **(d)** within the active site of ‘human erythrocyte catalase’ (PDB ID: 1DGH).

To bridge the gap between the observed chemical antioxidant capacity and potential biological relevance, an *in silico* docking analysis was carried out using Human Erythrocyte Catalase (PDB ID: 1DGH) as a biological target. Unlike chemical scavenging assays, this analysis explores the potential interaction of the botanical drug’s metabolites with a key endogenous antioxidant enzyme. The results illustrated in Table 3 show that the majority of docked metabolites exhibited binding affinities ranging from −5.6 to −7.9 kcal/mol. Notably, alloaromadendrene emerged as the most promising ligand, with a docking score of −7.9 kcal/mol, which surpassed that of the reference standard, Vitamin E (−7.7 kcal/mol). The 3D model (Figure 3) demonstrates that alloaromadendrene fits effectively within the binding cavity of the targeted enzyme. This stability is primarily mediated by a network of hydrophobic interactions, specifically Alkyl and Pi-Alkyl contacts with the amino acid residues: Arg72, Val74, His75, Val146, Phe334, Tyr158, and His362. Detailed intermolecular contacts for other docked molecules are provided in Figure 4. While direct docking between alloaromadendrene and human catalase has not been previously reported, our findings align with recent studies identifying this metabolite as a primary ligand for other therapeutic targets such as CYP2C9, xanthine oxidase, and calpain-1 (Minchán-Herrera et al., 2022). These insights suggest that the metabolites of *A. lanatum* essential oil may interact with endogenous antioxidant systems, providing a theoretical molecular basis for the scavenging effects observed in the analytical assays.

**TABLE 3:** Binding energy of the docked metabolites within the active site Human Erythrocyte Catalase’ (PDB ID: 1DGH).

Metabolites	Binding energy (kcal/mol)
Alloaromadendrene	−7.9
Longifolene	−5.6
(−)-Globulol	−6.8
*β*-asarone	−6.4
Isopulegol	−6.3
Vitamin E (standard)	−7.7

#### 3.6.2 Docking to *α*–amylase target

**FIGURE 5:**
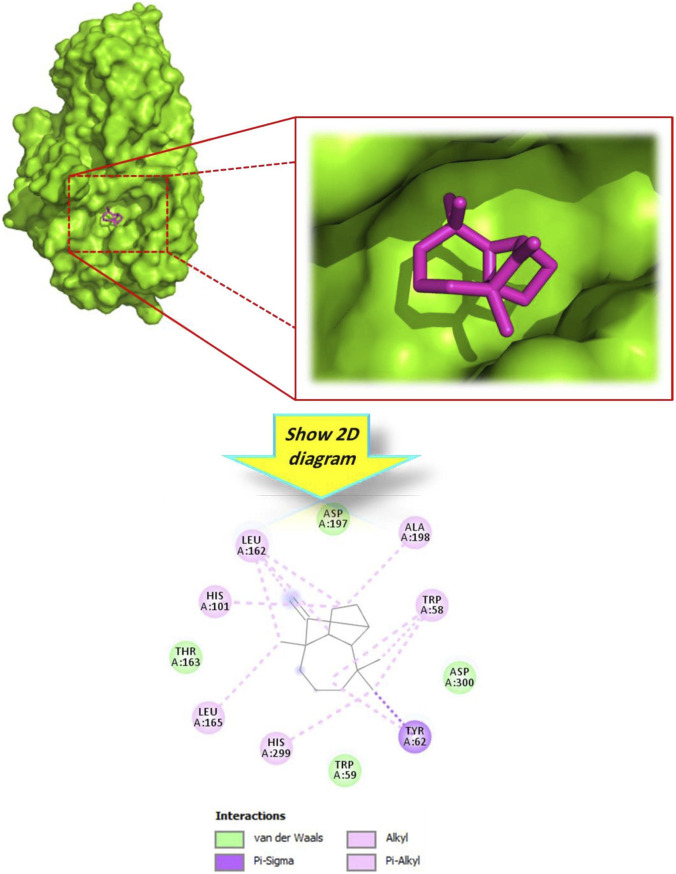
3D and 2D interactions model of the most active metabolite ‘longifolene’ within the active site of ‘a-amylase’ (PDB ID: 3BAJ).

**FIGURE 6:**
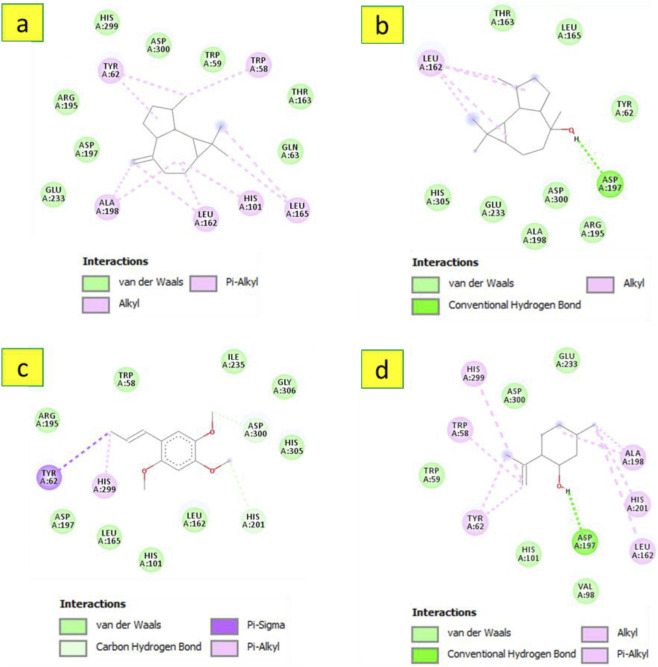
2D model of different interactions formed by the docked metabolites: alloaromadendrene **(a)**, (−)-globulol **(b)**, B-asarone **(c)**, and isopulegol **(d)** within the active site of ‘a-amylase’ (PDB ID: 3BAJ).

Concerning *α*-amylase (PDB ID: 3BAJ), the data presented in Table 4 indicate that longifolene was theoretically found to be the most active metabolite (binding energy = −7 kcal/mol) followed by alloaromadendrene (binding energy = −6.9 kcal/mol). These values are comparable to the reference, acarbose, which exhibited a binding energy of - 7.4 kcal/mol. Longifolene fits well in the active site of the targeted enzyme, as shown in Figure 5, involved in a Pi-Sigma interaction with Tyr62 and several Alkyl/Pi-Alkyl contacts with Trp58, Tyr62, His101, Leu162, Leu165, Ala198, and His299. Regarding the other metabolites (alloaromadendrene, (−)-globulol, *β*-asarone, and isopulegol), they established several interactions as shown in Figure 6. The search for natural carbohydrate-digesting enzyme inhibitors to control postprandial hyperglycemia has validated *α*-amylase as a pharmacological target. The inhibition of *α*-amylase activity leads to reduced starch breakdown and glucose absorption, which helps patients with type 2 diabetes.

**TABLE 4:** Binding energy of the docked metabolites in the binding cavity of ‘α–amylase’ (PDB: 3BAJ).

Metabolites	Binding energy (kcal/mol)
Alloaromadendrene	−6.9
Longifolene	−7.0
(−)-Globulol	−5.8
*β*-asarone	−4.9
Isopulegol	−5.4
Acarbose (standard)	−7.4

The inhibition of *α*-amylase activity is known to reduce starch breakdown and subsequent glucose absorption, thereby contributing to glycemic control in patients with type 2 diabetes. In this context, *in silico* molecular docking approaches are increasingly employed as predictive and hypothesis-generating tools for screening plant-derived metabolites with potential *α*-amylase inhibitory activity. Previous docking studies on essential oil metabolites from various botanical drugs have reported correlations between predicted binding affinities and experimental inhibition data (Tolmie et al., 2021); however, such computational findings remain theoretical approximations rather than definitive proof of biological activity. To date, the literature provides limited information regarding longifolene and alloaromadendrene as potential *α*-amylase-binding metabolites. Although these sesquiterpene hydrocarbons exhibit structural features that may favor enzyme interaction, the present study offers only a computational prediction of their binding behavior, not an experimental confirmation of inhibitory activity. The findings suggest that sesquiterpene hydrocarbons could represent a promising avenue for the exploration of novel antidiabetic natural metabolites targeting *α*-amylase; nevertheless, this assumption should be interpreted cautiously. Our results expand the structural landscape of natural metabolites investigated for glucose regulation by proposing a putative binding mode of longifolene and alloaromadendrene within the *α*-amylase active site. Importantly, these observations are preliminary and based solely on docking simulations. Therefore, the predicted interactions and binding affinities require rigorous experimental validation. Further *in vitro α*-amylase inhibition assays are essential to confirm whether the computationally predicted interactions translate into measurable biological activity.

#### 3.6.3 Docking to anti-lipoxygenase target

**FIGURE 7:**
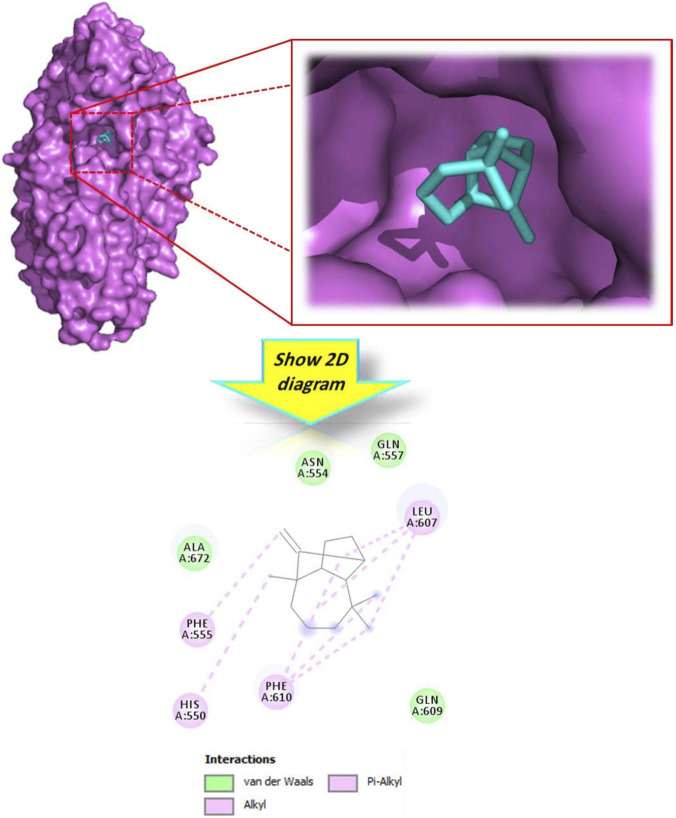
3D and 2D interactions model of the most active metabolite ‘longifolene’ within the active site of ‘lipoxygenase’ (PDB ID: 3V99).

**FIGURE 8:**
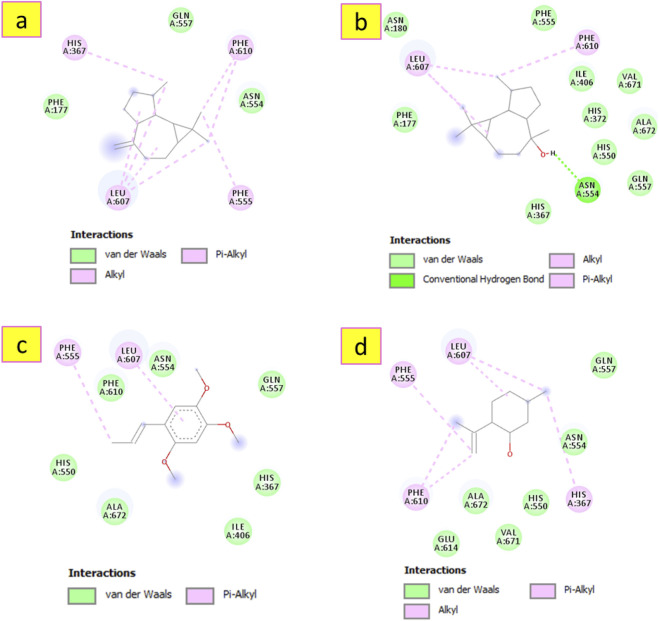
2D model of different interactions formed by the docked metabolites: alloaromadendrene **(a)**, (−)-globulol **(b)**, ß-asarone **(c)** and isopulegol **(d)** within the active site of ‘lipoxygenase’ (PDB ID: 3V99).

Concerning the docking complex: ‘lipoxygenase’-phytoligands, as depicted in Table 5, theoretically, ‘longifolene’ was also found to be the most bioactive ligand compared to its analogs. As illustrated in Figure 7, this metabolite fits well into the active site of the target receptor. Specifically, it exhibited alkyl and Pi-alkyl interactions with key residues including His550, Phe555, Leu607, and Phe610. Figure 8 further demonstrates that the other docked molecules also formed interactions with residues within the active site. The docking analysis of the lipoxygenase target (PDB ID: 3V99) indicated that the main sesquiterpenes from the extract bind to the enzyme with acceptable strength. For comparison purposes, longifolene (−6.3 kcal/mol) and alloaromadendrene (−6.1 kcal/mol) showed the most favorable predicted binding energies among the tested natural metabolites, followed by (−)-globulol (−5.9 kcal/mol). These values were higher than those obtained for *β*-asarone (−4.8 kcal/mol) and isopulegol (−5.0 kcal/mol). In comparison, dexamethasone exhibited stronger predicted binding to the enzyme than longifolene and alloaromadendrene. However, their comparable binding scores suggest that these metabolites may represent promising candidates for further investigation. There is limited research on sesquiterpene hydrocarbons as LOX inhibitors. The present findings provide preliminary computational insights into the possible interactions of these metabolites with the lipoxygenase target. Further experimental studies are required to validate their predicted LOX-inhibitory potential.

**TABLE 5:** Binding energy of the docked metabolites in the binding cavity of ‘lipoxygenase’ (PDB: 3V99).

Metabolites	Binding energy (kcal/mol)
Alloaromadendrene	−6.1
Longifolene	−6.3
(−)-Globulol	−5.9
*β*-asarone	−4.8
Isopulegol	−5.0
Dexamethasone (standard)	−8.2

#### 3.6.4 Docking to antibacterial target

**FIGURE 9:**
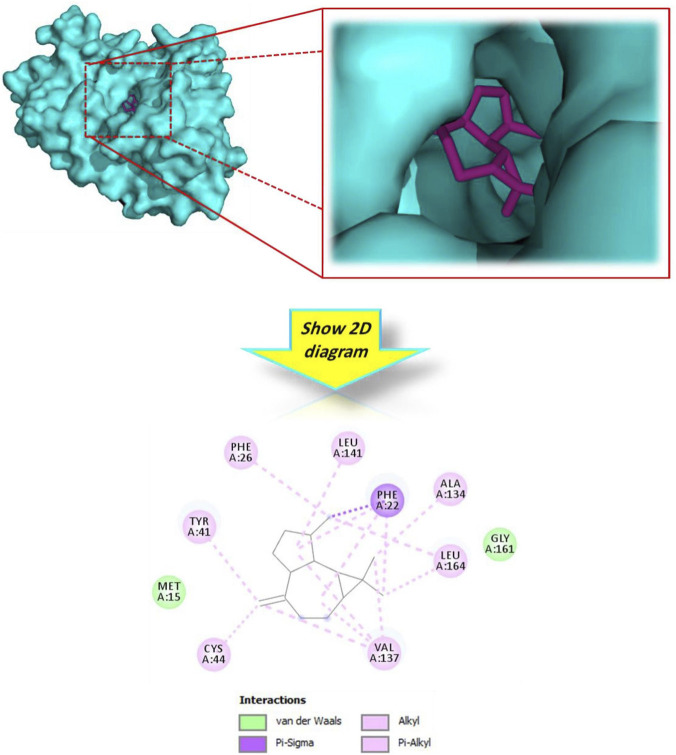
3D and 2D interactions model of the most active metabolite ‘alloaromadendrene’ within the active site of *S. aureus* dehydrosqualene synthase’ (PDB ID: 2ZCQ).

**FIGURE 10:**
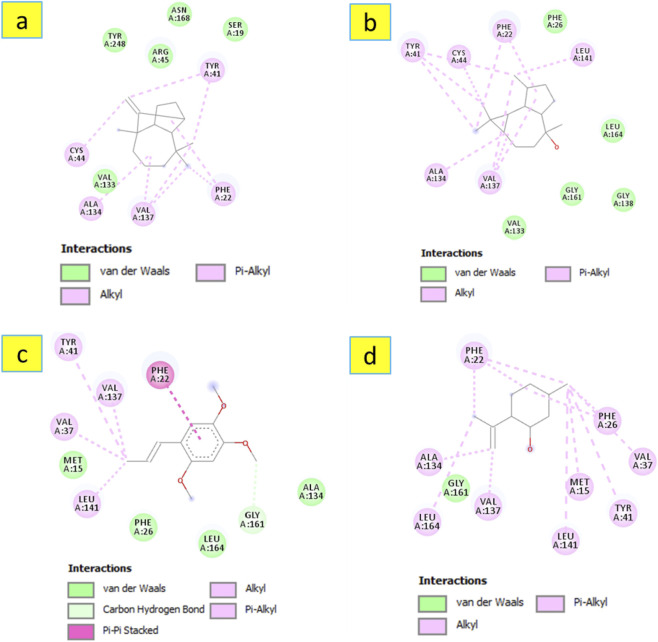
2D model of different interactions formed by the docked metabolites: longifolene **(a)**, (−)-globulol **(b)**, ẞ-asarone **(c)** and isopulegol **(d)** within the active site of *S. aureus* dehydrosqualene synthase’ (PDB ID: 2ZCQ).

Regarding the docking complex of *S. aureus* dehydrosqualene synthase (CrtM) and the investigated metabolites, the docking simulation proposes a putative binding conformation that may be consistent with the hypothesized antibacterial mechanism. The results of this modeling approach, shown in Table 6, indicate that most docked metabolites exhibited binding scores ranging from −6.5 to −8.7 kcal/mol. These predicted binding affinities suggest possible interactions with the enzyme that may be relevant for its inhibition, pending further validation. Notably, alloaromadendrene demonstrated the most favorable predicted docking performance, with a binding energy of −8.9 kcal/mol, which is more favorable than that of the reference metabolite, ciprofloxacin. The 3D docking model in Figure 9 shows that alloaromadendrene fits well within the enzyme’s active site, forming multiple hydrophobic interactions, including π-Sigma interaction with Phe22, and alkyl and π–Alkyl interactions with residues Phe22, Phe26, Tyr41, Cys44, Ala134, Val137, Leu141, and Leu164. Additionally, other docked metabolites also establish several intermolecular interactions, as illustrated in Figure 10. Molecular docking was performed to explore how sesquiterpenes such as alloaromadendrene, longifolene, globulol, *β*-asarone, and isopulegol may interact with the *S*. *aureus* dehydrosqualene synthase (CrtM) enzyme (PDB ID: 2ZCQ), with the aim of providing insights into their possible mechanism of interaction with this target. The 2ZCQ structure includes CrtM, which catalyzes staphyloxanthin biosynthesis. Docking of essential oil sesquiterpenes to the CrtM enzyme has been proposed as an approach to identify metabolites that may interfere with pigment production and reduce bacterial resistance to oxidative stress (Lin et al., 2010). The present results indicate that alloaromadendrene, along with longifolene and globulol, which are major metabolites of *A. lanatum* essential oil and was show favorable predicted interactions in the docking simulations.

**TABLE 6:** Binding energy of the docked metabolites in the binding cavity of ‘*S. aureus* dehydrosqualene synthase’ (PDB: 2ZCQ).

Metabolites	Binding energy (kcal/mol)
Alloaromadendrene	−8.7
Longifolene	−7.3
(−)-Globulol	−8.6
*β*-asarone	−6.6
Isopulegol	−6.5
Ciprofloxacin (standard)	−6.7

### 3.7 Pharmacokinetic and ADME properties

**FIGURE 11:**
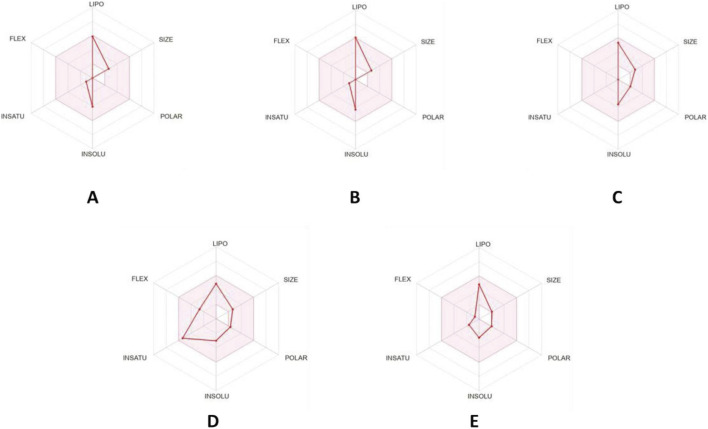
Bioavailability radar of the selected metabolites: **(A)** alloaromadendrene, **(B)** longifolene, **(C)** (−)-globulol, **(D)** ẞ-asarone and **(E)** isopulegol.

**FIGURE 12:**
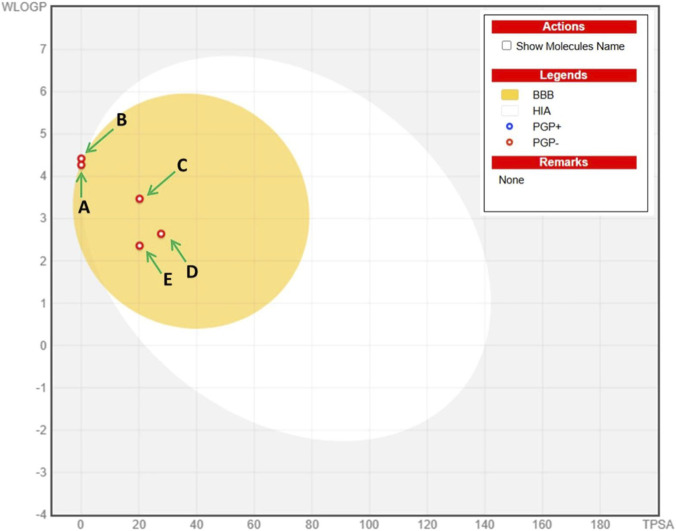
Boiled-egg graph of the selected metabolites: **(A)** alloaromadendrene, **(B)** longifolene, **(C)** (−)-globulol, **(D)** ẞ-asarone and **(E)** isopulegol.

The forecasting of ADME (absorption, distribution, metabolism, and excretion) data of the major metabolites has been estimated to provide insights into their pharmacokinetic properties. The predicted descriptors including their pharmacokinetic and drug-likeness properties, are illustrated in Table 7. All tested ligands were found to meet the rules of Lipinski, Veber and Egan, and share topological polar surface area (TPSA) values ranging from 00.00 to 27.69Å^2^, suggesting possible favorable passive oral absorption, as reflected by the consensus Log Po/w in the range 2.44–4.50. Furthermore, a bioavailability score of 0.55 indicates favorable predicted oral bioavailability. As noted in Table 7, none of the metabolites were predicted to be P-glycoprotein (P–gp) substrates, suggesting possible favorable intestinal absorption and bioavailability. The tested ligands, including (−)-globulol, *β*-asarone, and isopulegol, displayed high predicted gastrointestinal absorption (GI), and alloaromadendrene, along with these three metabolites, was predicted to cross the blood–brain barrier (BBB). Most metabolites showed no inhibitory effect on the main cytochrome (CYP 450) enzymes: CYP1A2, CYP2C19, CYP2C9, CYP2D6, and CYP3A4. The radar plot (Figure 11) shows that all tested molecules are located within the pink zone, indicating their potential for better drug-likeness and a good bioavailability profile. On the other hand, Figure 12 presents the BOILED-EGG model, which is used to predict gastrointestinal absorption (HIA) and BBB penetration. This model defines two regions: one corresponding to GI absorption (HIA) and the other to BBB penetration (yolk). Neither ‘GI absorption’ nor ‘BBB penetration’ is indicated if any metabolite is found in the gray zone. According to the results, four out of five metabolites appear in the yellow region (yolk), with red points indicating their high probability of brain penetration (BB) and acting as non-substrate behavior for P–gp. The five main metabolites showed predicted drug-like properties based on ADME profiling, as all metabolites met the Lipinski, Veber, and Egan criteria and shared a bioavailability scores of 0.55. Nevertheless, *β*-asarone, present at 11.27%, is a well-documented genotoxic and carcinogenic metabolite (Chellian et al., 2017). Its high predicted GI absorption and BBB permeability may represent a potential safety concern rather than a therapeutic advantage. Thus, while the essential oil exhibits promising pharmacokinetic characteristics, further dose-dependent toxicological evaluations are necessary before any therapeutic application can be considered. The metabolites showed suitable predicted drug-like properties, meeting the requirements for molecular weight, polarity, and structural flexibility associated with oral drug development. The sesquiterpenes longifolene and alloaromadendrene showed poor predicted GI absorption; however, their high lipophilicity may facilitate their ability to cross the blood-brain barrier, suggesting potential access to the central nervous system despite limited intestinal absorption. In contrast, the oxygenated monoterpenes and phenylpropanoids demonstrated both high predicted GI absorption and BBB permeability, likely due to a balanced polarity–lipophilicity ratio. The metabolites were not predicted to be P-gp substrates, which may be favorable in avoiding reduced bioavailability due to efflux mechanisms. The CYP-inhibition results indicated specific predicted interactions, as several metabolites were suggested to inhibit CYP1A2 or CYP2C19, which could lead to metabolism-related pharmacokinetic variations. The Log Kp values for skin permeation suggested that these lipophilic metabolites may exhibit moderate to high permeability. Overall, the tested metabolites suggest drug-like properties based on their ADME profiles, however, individual metabolites exhibit distinct characteristics that may influence their biological properties. This study provides a structural framework that may guide subsequent experimental investigations.

**TABLE 7:** *In silico* ADME analysis of the major metabolites.

Entry	A	B	C	D	E
GI absorption*	Low	Low	High	High	High
P–gp substrate*	No	No	No	No	No
BBB permeant*	Yes	No	Yes	Yes	Yes
CYP1A2 inhibitor*	Yes	No	No	Yes	No
CYP2C19 inhibitor*	Yes	Yes	Yes	Yes	No
CYP2C9 inhibitor*	Yes	Yes	No	No	No
CYP2D6 inhibitor*	No	No	No	No	No
CYP3A4 inhibitor*	No	No	No	No	No
Log Kp (cm/s)^a*^	−4.20	−3.94	−5.00	−5.44	−5.15
Lipinski**	Yes	Yes	Yes	Yes	Yes
Veber**	Yes	Yes	Yes	Yes	Yes
Egan**	Yes	Yes	Yes	Yes	Yes
Bioavailability score**	0.55	0.55	0.55	0.55	0.55
TPSA (Å^2^)***	00.00	00.00	20.23	27.69	20.23
Consensus Log *P*o/w****	4.34	4.50	3.41	2.69	2.44

A: alloaromadendrene; B: longifolene; C:(−)-Globulol; D: *β*-Asarone; E: isopulegol.

## 4 Conclusion

In conclusion, our findings show that the essential oil of *A. lanatum* growing wild in the Hail region possesses a notable chemical antioxidant capacity, along with significant biological effects, including *α*-amylase and lipoxygenase inhibition, as well as bactericidal effects against *Staphylococcus aureus.* Phytochemical and analytical characterization revealed a complex profile of oxygenated and hydrocarbon terpenes, which are primarily responsible for the observed radical scavenging potential. *In silico* docking and ADME characteristics of the major metabolites, such as isopulegol and alloaromadendrene, provide a theoretical molecular basis for their interactions with key biological targets (PDB: 1DGH, 3BAJ, 3V99, and 2ZCQ). While the oil exhibits promising pharmacological potential in terms of enzyme inhibition and antimicrobial properties, its chemical antioxidant capacity serves as a foundational analytical indicator of its redox properties. Further safety evaluations and *in vivo* dose-dependent assessments are recommended to validate these preliminary findings before considering any therapeutic applications.

## Data Availability

The original contributions presented in the study are included in the article/Supplementary Material, further inquiries can be directed to the corresponding author.

## References

[B1] Adams R. P. (2017). Identification of essential oil components by gas chromatography/mass spectrometry. 5th online ed. Nashville, TN, USA: Texensis Publishing.

[B2] Al Kury L. T. Abdoh A. Ikbariah K. Sadek B. Mahgoub M. (2021). *In vitro* and *in vivo* antidiabetic potential of monoterpenoids: an update. Mol. Basel, Switz. 27 (1), 182. 10.3390/molecules27010182 35011414 PMC8746715

[B3] Al-Mazroa S. (2003). Chemical constituents of *A. lanatum, H. tuberculatum, A. garcini, S. spinosa, H. bacciferum*, and *A. ludwigii* grown in Saudi Arabia. J. Saudi Chem. 7, 255–258.

[B4] Al-Mijalli S. H. Mrabti H. N. Abdallah E. M. Assaggaf H. Qasem A. Alenazy R. (2025). *Acorus calamus* as a promising source of new antibacterial agent against *Pseudomonas aeruginosa* and *Staphylococcus aureus*: deciphering volatile compounds and mode of action. Microb. Pathogenesis 200, 107357. 10.1016/j.micpath.2025.107357 39894234

[B5] Ali H. Houghton P. J. Soumyanath A. (2006). alpha-Amylase inhibitory activity of some Malaysian plants used to treat diabetes; with particular reference to *Phyllanthus amarus* . J. Ethnopharmacology 107 (3), 449–455. 10.1016/j.jep.2006.04.004 16678367

[B6] Amiri M. S. Joharchi M. R. (2016). Ethnobotanical knowledge of Apiaceae family in Iran: a review. Avicenna J. Phytomed. 6, 621–635. 28078243 PMC5206921

[B7] Arraji M. Al Wachami N. Boumendil K. Chebabe M. Mochhoury L. Laamiri F. Z. (2024). Ethnobotanical survey on herbal remedies for the management of type 2 diabetes in the Casablanca-Settat region, Morocco. BMC Complementary Med. Ther. 24, 160. 10.1186/s12906-024-04468-4 38622669 PMC11017650

[B8] Ayadi J. Debouba M. Rahmani R. Bouajila J. (2023). The phytochemical screening and biological properties of *Brassica napus* L. var. *napobrassica* (Rutabaga) seeds. Mol. Basel, Switz. 28 (17), 6250. 10.3390/molecules28176250 37687079 PMC10488400

[B9] Bag S. Chatterjee D. Ashique S. Pal R. Khatoon H. Kumar S. (2024). Antidiabetic potential of Apiaceae family plants – a critical update. Curr. Diabetes Rev. 20 (2), e290224227538. 10.2174/0126668629283987240123100449

[B10] Ben Selma W. Alibi S. Ferjeni M. Ghezal S. Gallala N. Belghouthi A. (2024a). Synergistic activity of *Thymus capitatus* essential oil and cefotaxime against ESBL-producing *Klebsiella pneumoniae* . Int. Journal Environmental Health Research 34 (8), 2936–2946. 10.1080/09603123.2023.2280149 37952172

[B11] Ben Selma W. Farouk A. Ban Z. Ferjeni M. Alsulami T. Ali H. (2024b). *Thymus algeriensis* essential oil: phytochemical investigation, bactericidal activity, synergistic effect with colistin, molecular docking, and dynamics analysis against Gram-negative bacteria resistant to colistin. Heliyon 10 (19), e38281. 10.1016/j.heliyon.2024.e38281 39386781 PMC11461995

[B12] Ben Selma W. Ferjeni S. Farouk A. Marzouk M. Boukadida J. (2025). Antimicrobial activity of *Cinnamomum zeylanicum* essential oil against colistin-resistant gram-negative bacteria. Int. Journal Environmental Health Research 35 (1), 169–181. 10.1080/09603123.2024.2348094 38695857

[B13] Besbes M. Omri A. Cheraif I. Daami M. Jannet H. B. Mastouri M. (2012). Chemical composition and antimicrobial activity of essential oils from *Scabiosa arenaria* Forssk: growing wild in Tunisia. Chem. & Biodiversity 9 (4), 829–839. 10.1002/cbdv.201100191 22492499

[B14] Besbes M. Hamdi A. Horchani M. Majouli K. Ghorbel M. Lotfi S. (2025a). Phytochemical screening, phytotoxic effects and *in silico* studies of *Zilla Spinosa* L. and *Farsetia Aegyptia* turra extracts growing in hail region. J. Soil Sci. Plant Nutr. 25, 2052–2069. 10.1007/s42729-025-02256-8

[B15] Besbes M. Hamdi A. Chahdoura H. Alshammari A. A. Al-Shammari W. B. AlArdan D. (2025b). The genus *Anisosciadium* **:** a comprehensive review of taxonomic aspects, traditional uses, phytochemistry, and biological activities. Processes 13, 2475. 10.3390/pr13082475

[B16] Besbes M. Hamdi A. Horchani M. Majouli K. Dbeibia A. Jilani J. (2026). Phytochemical profiling, antimicrobial, antibiofilm, and molecular docking of *Farsetia aegyptia* and *Zilla spinosa* from Saudi Arabia. Front. Pharmacol. 16: 1723207. 10.3389/fphar.2025.1723207 41710549 PMC12910216

[B17] Chellian R. Pandy V. Mohamed Z. (2017). Pharmacology and toxicology of α- and β-Asarone: a review of preclinical evidence. Phytomedicine 32, 41–58. 10.1016/j.phymed.2017.04.003 28732807

[B18] Chen X. Xie N. Feng L. Huang Y. Wu Y. Zhu H. (2025). Oxidative stress in diabetes mellitus and its complications: from pathophysiology to therapeutic strategies. Chin. Med. J. 138 (1), 15–27. 10.1097/CM9.0000000000003230 39503316 PMC11717531

[B27] Cherneva D. Mihalev K. Iliev I. Agova N. Yaneva G. Dimitrova T. (2025). *In silico* evaluation of terpene interactions with inflammatory enzymes: a blind docking study targeting arachidonic acid metabolism. Appl. Sci. 15 (13), 7536. 10.3390/app15137536

[B19] Cirino I. C. D. S. de Santana C. F. Bezerra M. J. R. Rocha I. V. Luz A. C. O. Coutinho H. D. M. (2023). Comparative transcriptomics analysis of multidrug-resistant *Acinetobacter baumannii* in response to treatment with the terpenic compounds thymol and carvacrol. Biomed. & Pharmacotherapy = Biomedecine & Pharmacotherapie 165, 115189. 10.1016/j.biopha.2023.115189 37481932

[B20] Clinical and Laboratory Standards Institute (CLSI) (2019). Performance standards for antimicrobial susceptibility testing. 29th ed. CLSI Suppl M100. Wayne, PA: Clinical and Laboratory Standards Institute. 10.1007/978-3-662-48986-4

[B21] Delano W. L. (2002). The PyMOL molecular graphics system. Version 0.99rc6. De Lano Scientific, San Carlos: Schrödinger, LLC.

[B22] Edziri H. Mastouri M. Mahjoub M. A. Mighri Z. Mahjoub A. Verschaeve L. (2012). Antibacterial, antifungal and cytotoxic activities of two flavonoids from *Retama raetam* flowers. Mol. Basel, Switz. 17 (6), 7284–7293. 10.3390/molecules17067284 22695233 PMC6268215

[B23] El-Sayed W. M. Hussin W. A. Mahmoud A. A. AlFredan M. A. (2017). Antimutagenic activities of *Anisosciadium lanatum* extracts could predict the anticancer potential in different cell lines. Int. J. Pharm. Pharm. Res. 9, 197–206. 10.25258/phyto.v9i2.8063

[B24] Eshwarappa R. S. Ramachandra Y. L. Subaramaihha S. R. Subbaiah S. G. Austin R. S. Dhananjaya B. L. (2016). Anti-lipoxygenase activity of leaf gall extracts of *Terminalia chebula* (Gaertn.) retz. (Combretaceae). Pharmacogn. Research 8 (1), 78–82. 10.4103/0974-8490.171103 26941541 PMC4753765

[B25] EUCAST (2024). Clinical breakpoint (v 14.0). Available online at: https://www.eucast.org/fileadmin/src/media/PDFs/EUCAST_files/Breakpoint_tables/v_14.0_Breakpoint_Tables.pdf (Accessed December 27, 2024).

[B26] Gadnayak A. Dehury B. Nayak A. Jena S. Sahoo A. Panda P. C. (2022). 'Mechanistic insights into 5-lipoxygenase inhibition by active principles derived from essential oils of Curcuma species: molecular docking, ADMET analysis and molecular dynamic simulation study. PloS One 17 (7), e0271956. 10.1371/journal.pone.0271956 35867724 PMC9307165

[B28] Gilbert N. C. Rui Z. Neau D. B. Waight M. T. Bartlett S. G. Boeglin W. E. (2012). “Conversion of human 5-lipoxygenase to a 15-lipoxygenase by a point mutation to mimic phosphorylation at Serine-663,”, 26. FASEB journal: official publication of the Federation of American Societies for Experimental Biology, 3222–3229. 10.1096/fj.12-205286 22516296 PMC3405276

[B29] Hamdi A. Horchani M. Jannet H. B. Snoussi M. Noumi E. Bouali N. (2023). *In vitro* screening of antimicrobial and anti-coagulant activities, ADME profiling, and molecular docking study of *citrus limon* L. and *Citrus paradisi* L. cold-pressed volatile oils. Pharm. Basel, Switz. 16 (12), 1669. 10.3390/ph16121669 38139796 PMC10748103

[B30] Heinrich M. Jalil B. Abdel-Tawab M. Echeverria J. Kulić Ž. McGaw L. J. (2022). Best practice in the chemical characterisation of extracts used in pharmacological and toxicological research—The ConPhyMP—Guidelines1fnfn32fnfn4. Front. Pharmacol. 13, 953205. 10.3389/fphar.2022.953205 36176427 PMC9514875

[B31] Hlila M. B. Mosbah H. Mssada K. Jannet H. B. Aouni M. Selmi B. (2015). Acetylcholinesterase inhibitory and antioxidant properties of roots extracts from the Tunisian *Scabiosa arenaria* forssk. Industrial Crops Prod. 67, 62–69. 10.1016/j.indcrop.2015.01.009

[B32] Hlila M. B. Mosbah H. Zanina N. Ben Nejma A. Ben Jannet H. Aouni M. (2016). Characterisation of phenolic antioxidants in *Scabiosa arenaria* flowers by LC-ESI-MS/MS and NMR. J. Pharmacy Pharmacology 68 (7), 932–940. 10.1111/jphp.12561 27230582

[B33] Iskandar K. Ahmed N. Paudyal N. Ruiz Alvarez M. J. Balasubramani S. P. Saadeh D. (2025). Essential oils as antimicrobial agents against WHO priority bacterial pathogens: a strategic review of *in vitro* clinical efficacy, innovations and research gaps. Antibiotics 14 (12), 1250. 10.3390/antibiotics14121250 41463752 PMC12729739

[B34] Jilani S. Ferjeni M. Al-Shammery K. Rashid Mohammed AlTamimi H. Besbes M. Ahmed Lotfi S. (2025). The synergistic effect of *Thymus vulgaris* essential oil and carvacrol with imipenem against carbapenem-resistant *Acinetobacter baumannii: in vitro,*, molecular docking, and molecular dynamics studies. Front. Pharmacology 16, 1582102. 10.3389/fphar.2025.1582102 40474976 PMC12137262

[B35] Jilani S. Mohammed AlTamimi H. R. Bayar S. Lotfi S. A. Besbes M. Al-Shammery K. (2026). Synergistic antibacterial effects of clove essential oil and eugenol with ciprofloxacin against MDR Gram-negative bacteria: *in vitro* and *in silico* approaches. S. Afr. J. Bot. 190, 567–580. 10.1016/j.sajb.2026.02.001

[B36] Khalil H. E. Ibrahim H. M. Darrag H. M. Matsunami K. (2022). Insight into analysis of essential oil from *Anisosciadium lanatum* Boiss.-Chemical composition, molecular docking, and mitigation of Hepg2 cancer cells through apoptotic markers. Plants Basel, Switz. 11 (1), 66. 10.3390/plants11010066 35009072 PMC8747166

[B37] Lim H. Lee S. Y. Lee K. R. Kim Y. S. Kim H. P. (2012). The rhizomes of Acorus gramineus and the constituents inhibit allergic response *in vitro* and *in vivo* . Biomol. & Therapeutics 20 (5), 477–481. 10.4062/biomolther.2012.20.5.477 24009837 PMC3762279

[B38] Lin F. Y. Liu C. I. Liu Y. L. Zhang Y. Wang K. Jeng W. Y. (2010). Mechanism of action and inhibition of dehydrosqualene synthase. Proc. Natl. Acad. Sci. U. S. A. 107 (50), 21337–21342. 10.1073/pnas.1010907107 21098670 PMC3003041

[B39] Liu C. I. Liu G. Y. Song Y. Yin F. Hensler M. E. Jeng W. Y. (2008). A cholesterol biosynthesis inhibitor blocks *Staphylococcus aureus* virulence. Sci. (New York, N.Y.) 319 (5868), 1391–1394. 10.1126/science.1153018 18276850 PMC2747771

[B40] Ma Z. Cao X. Guo X. Wang M. Ren X. Dong R. (2018). Establishment and validation of an *in vitro* screening method for traditional Chinese medicine-induced nephrotoxicity. Evidence-based Complementary Alternative Medicine eCAM 2018, 2461915. 10.1155/2018/2461915 30050583 PMC6046169

[B41] Mahmoud A. A. El-Sayed W. M. (2019). The anti-proliferative activity of anisosciadone: a new guaiane sesquiterpene from *Anisosciadium lanatum* . Anti-cancer Agents Medicinal Chemistry 19 (9), 1114–1119. 10.2174/1871520619666190308112732 30848216

[B42] Mandaville J. P. (2019). Bedouin ethnobotany: plant concepts and uses in a desert pastoral world. Tucson, AZ: University of Arizona Press.

[B43] Masroorbabanari M. Miri R. Firuzi O. Jassbi A. R. (2014). Antioxidant and cytotoxic activities of the essential oil and extracts of *Anisosciadium orientale* . J. Chem. Soc. Pak. 36, 457–461.

[B44] Matar M. W. Alqahtani S. M. N. Alghafli D. A. Altaweel A. A. Alasoom A. J. Burshed H. A. (2022). Phytochemical approach including total phenolic and flavonoid contents and evaluation of *in vitro* ABTS antioxidant capacity and lipoxygenase inhibition of *Anisosciadium lanatum* . Pharmacogn. J. 14, 928–932. 10.5530/pj.2022.14.191

[B45] Maurus R. Begum A. Williams L. K. Fredriksen J. R. Zhang R. Withers S. G. (2008). Alternative catalytic anions differentially modulate human alpha-amylase activity and specificity. Biochemistry 47 (11), 3332–3344. 10.1021/bi701652t 18284212

[B46] Minchán-Herrera P. Ybañez-Julca R. O. Quispe-Díaz I. M. Venegas-Casanova E. A. Jara-Aguilar R. Salas F. (2022). *Valeriana pilosa* roots essential oil: Chemical composition, antioxidant activities, and molecular docking studies on enzymes involved in Redox biological processes. Antioxidants Basel, Switz. 11 (7), 1337. 10.3390/antiox11071337 35883828 PMC9311991

[B47] Muilu-Mäkelä R. Aapola U. Tienaho J. Uusitalo H. Sarjala T. (2022). Antibacterial and oxidative stress-protective effects of five monoterpenes from Softwood. Mol. Basel, Switz. 27 (12), 3891. 10.3390/molecules27123891 35745011 PMC9230896

[B48] Mulyaningsih S. Sporer F. Zimmermann S. Reichling J. Wink M. (2010). Synergistic properties of the terpenoids aromadendrene and 1,8-cineole from the essential oil of *Eucalyptus globulus* against antibiotic-susceptible and antibiotic-resistant pathogens. Phytomedicine International Journal Phytotherapy Phytopharmacology 17 (13), 1061–1066. 10.1016/j.phymed.2010.06.018 20727725

[B49] Naigre R. Kalck P. Roques C. Roux I. Michel G. (1996). Comparison of antimicrobial properties of monoterpenes and their carbonylated products. Planta Medica. 62 (3), 275–277. 10.1055/s-2006-957877 8693045

[B50] Nazir A. Nazir A. Zuhair V. Aman S. Sadiq S. U. R. Hasan A. H. (2025). The global challenge of antimicrobial resistance: mechanisms, case studies, and mitigation approaches. Health Sci. Rep. 8 (7), e71077. 10.1002/hsr2.71077 40704322 PMC12284435

[B51] Nazzaro F. Fratianni F. De Martino L. Coppola R. De Feo V. (2013). Effect of essential oils on pathogenic bacteria. Pharm. Basel, Switz. 6 (12), 1451–1474. 10.3390/ph6121451 24287491 PMC3873673

[B52] Nile S. H. Nile A. Oh J.-W. Kai G. (2020). Soybean processing waste: potential antioxidant, cytotoxic and enzyme inhibitory activities. Food Biosci. 38, Article 100778. 10.1016/j.fbio.2020.100778

[B53] Önder S. Periz Ç. D. Ulusoy S. Erbaş S. Önder D. Tonguç M. (2024). Chemical composition and biological activities of essential oils of seven cultivated Apiaceae species. Sci. Reports 14 (1), 10052. 10.1038/s41598-024-60810-3 38698117 PMC11066118

[B54] Pankey G. A. Sabath L. D. (2004). Clinical relevance of bacteriostatic versus bactericidal mechanisms of action in the treatment of Gram-positive bacterial infections. Clin. Infectious Diseases An Official Publication Infect. Dis. Soc. Am. 38 (6), 864–870. 10.1086/381972 14999632

[B55] Pinzi L. Rastelli G. (2019). Molecular docking: shifting paradigms in drug discovery. Int. Journal Molecular Sciences 20 (18), 4331. 10.3390/ijms20184331 31487867 PMC6769923

[B56] POWO (2026). Plants of the world online. Kew: Facilitated by the Royal Botanic Gardens. Available online at: http://www.plantsoftheworldonline.org/(Accessed April 4, 2026).

[B57] Putnam C. D. Arvai A. S. Bourne Y. Tainer J. A. (2000). Active and inhibited human catalase structures: ligand and NADPH binding and catalytic mechanism. J. Mol. Biol. 296 (1), 295–309. 10.1006/jmbi.1999.3458 10656833

[B58] Redondo-Blanco S. Fernández J. López-Ibáñez S. Miguélez E. M. Villar C. J. Lombó F. (2020). Plant phytochemicals in food preservation: antifungal bioactivity: a review. J. Food Protection 83 (1), 163–171. 10.4315/0362-028X.JFP-19-163 31860394

[B59] Rijo P. Barros L. Efferth T. (2024). Editorial: III Bio. Natural-bioactive natural products research meeting: pharmacology perspectives. Front. Pharmacology 15, 1517673. 10.3389/fphar.2024.1517673 39629080 PMC11614142

[B60] Schmidt E. Bail S. Friedl S. M. Jirovetz L. Buchbauer G. Wanner J. (2010). Antimicrobial activities of single aroma compounds. Nat. Product Communications 5 (9), 1365–1368. 10.1002/cbdv.200900143 20922992

[B61] Shaw G. Morse S. Ararat M. Graham F. L. (2002). “Preferential transformation of human neuronal cells by human adenoviruses and the origin of HEK 293 cells,”, 16. FASEB journal : official publication of the Federation of American Societies for Experimental Biology, 869–871. 10.1096/fj.01-0995fje 11967234

[B62] Sher H. Aldosari A. (2013). Ethnobotanical Survey on plants of veterinary importance around Al-Riyadh (Saudi Arabia). Afr. J. Pharm. And Pharmacol. 7, 1404–1410. 10.5897/AJPP2012.2177

[B63] Souza P. M. Sales P. M. Simeoni L. A. Silva E. C. Silveira D. Magalhães P. deO. (2012). Inhibitory activity of α-amylase and α-glucosidase by plant extracts from the Brazilian cerrado. Planta Medica. 78 (4), 393–399. 10.1055/s-0031-1280404 22134849

[B64] Tarchouna M. Ferjani A. Ben-Selma W. Boukadida J. (2013). Distribution of uropathogenic virulence genes in Escherichia coli isolated from patients with urinary tract infection. Int. Journal Infectious Diseases IJID Official Publication Int. Soc. Infect. Dis. 17 (6), e450–e453. 10.1016/j.ijid.2013.01.025 23510539

[B65] Tolmie M. Bester M. J. Apostolides Z. (2021). Inhibition of α-glucosidase and α-amylase by herbal compounds for the treatment of type 2 diabetes: a validation of *in silico* reverse docking with *in vitro* enzyme assays. J. Diabetes 13 (10), 779–791. 10.1111/1753-0407.13163 33550683

[B66] Trott O. Olson A. J. (2010). AutoDock Vina: improving the speed and accuracy of docking with a new scoring function, efficient optimization, and multithreading. J. Comput. Chem. 31 (2), 455–461. 10.1002/jcc.21334 19499576 PMC3041641

[B67] Ullah H. Dacrema M. Buccato D. G. Fayed M. A. A. De Lellis L. F. Morone M. V. (2025). A narrative review on plant extracts for Metabolic syndrome: efficacy, safety, and technological advances. Nutrients 17 (5), 877. 10.3390/nu17050877 40077747 PMC11901876

[B68] US Food & Drug Administration (2024). Title 21, volume 3, 21CFR172.515. Regulations CoF.

[B69] WHO (2024). WHO bacterial priority pathogens list, 2024: bacterial pathogens of public health importance to guide research, development and strategies to prevent and control antimicrobial resistance. Available online at: https://iris.who.int/bitstream/handle/10665/376776/9789240093461-eng.pdf?sequence=1 (Accessed October 01, 2025).

[B70] Yesupatham A. Saraswathy R. (2025). Role of oxidative stress in prediabetes development. Biochem. Biophys. Rep. 43, 102069. 10.1016/j.bbrep.2025.102069 40519701 PMC12167118

