# Supplementary material for: Selective bioactive effects of Anisosciadium lanatum Boiss. [Apiaceae] essential oil: GC-MS profiling coupled with in Vitro and in silico screening
Source: Front Pharmacol. 2026 May 12;17:1772426. doi: 10.3389/fphar.2026.1772426 (PMC13201396; doi:10.3389/fphar.2026.1772426)
Supplement: Supplementary file 1 [file DataSheet1.pdf]

ConPhyMP Checklist Tables

Supplementary Table S1: ConPhyMP Checklist for *Anisosciadium lanatum* Boiss **essential Oil**

1	Authenic	Yes. Identified by Dr. Belsem Marzouk. The nomenclature was validated against the plants of the World Online (POWO) databases as *Anisosciadium lanatum* Boiss. Voucher specimen (Al- 83) is deposited at the herbarium of the laboratory of biology in the College of Sciences (Simira branch –Hail University, Saudi Arabia). Taxonomic authority and family [Apiaceae] are included (See Material and methods section, lines 102-108)
2	Well characterised	Yes. The phytochemical composition was established using Gas Chromatography-Mass Spectrometry (GC–MS) using a Clarus 600 T system. (See section, 2.3 Gas Chromatography –Mass Spectrometry analysis.
	Active ingredients known	Yes. Volatile phytocompounds were identified and discussed, the major bioactive constituents (e.g., isopulegol (22.39%), longifolene (19.73%), see Results and discussion section, 3.1 Chemical composition of the essential oil section pages 9-10),
	The chemical profile of the active ingredients/marker compounds is characterized qualitatively and quantitatively	Yes. Qualitative identification via Wiley/NIST libraries and retention indices (RI). Quantitative analysis based on peak area percentage. (See section, 2.3 Gas Chromatography –Mass Spectrometry analysis.
3	Free of adulteration and contamination	Yes. Extracted via hydrodistillation from fresh aerial parts; no solvents or additives were used.
4	Consistent	Yes. Extraction was performed under standardized conditions (100°C for 3h) to ensure reproducibility.
	Batch to batch variation is limited	Yes. The use of a standardized hydrodistillation protocol and fresh plant material ensured minimal variation between extraction batches, as confirmed by the reproducibility of the GC-MS chromatograms.
5	stable	Yes. Dried over anhydrous sodium sulfate and stored in amber glass vials at 4°C to prevent degradation. (See Section 2.2)

Supplementary Table S2a: Characterisation of the extraction process for *Anisosciadium lanatum* Boiss. essential oil (based on ConPhyMP guidelines)

**Parameter**	**Details of the Extraction Process**
**Plant Material**	Fresh aerial parts of *Anisosciadium lanatum* Boiss. (collected in March 2023, Hail, KSA).
**Extraction Method**	Hydrodistillation using a Clevenger-type apparatus.
**Solvent**	Distilled water (1.5 L).
**Solid/Liquid Ratio**	700 g of plant material in 1.5 L of distilled water.
**Extraction Temperature**	100 ± 2 °C.
**Extraction Duration**	3 hours.
**Pressure**	Atmospheric pressure.
**Drying/Collection**	Dried over anhydrous sodium sulfate (Na_2SO_4) and stored in amber vials at 4°C.
**Extraction Yield (%)**	0.035%
